# Crossover interference mechanism: New lessons from plants

**DOI:** 10.3389/fcell.2023.1156766

**Published:** 2023-05-19

**Authors:** Nahid Rafiei, Arnaud Ronceret

**Affiliations:** Plant Molecular Biology Department, Instituto de Biotecnología, Universidad Nacional Autónoma de México (UNAM), Cuernavaca, Mexico

**Keywords:** meiotic recombination, crossing-over, interference, synapsis, heterochiasmy, CO insurance, CO homeostasis, HEI10 coarsening model

## Abstract

Plants are the source of our understanding of several fundamental biological principles. It is well known that Gregor Mendel discovered the laws of Genetics in peas and that maize was used for the discovery of transposons by Barbara McClintock. Plant models are still useful for the understanding of general key biological concepts. In this article, we will focus on discussing the recent plant studies that have shed new light on the mysterious mechanisms of meiotic crossover (CO) interference, heterochiasmy, obligatory CO, and CO homeostasis. Obligatory CO is necessary for the equilibrated segregation of homologous chromosomes during meiosis. The tight control of the different male and female CO rates (heterochiasmy) enables both the maximization and minimization of genome shuffling. An integrative model can now predict these observed aspects of CO patterning in plants. The mechanism proposed considers the Synaptonemal Complex as a canalizing structure that allows the diffusion of a class I CO limiting factor linearly on synapsed bivalents. The coarsening of this limiting factor along the SC explains the interfering spacing between COs. The model explains the observed coordinated processes between synapsis, CO interference, CO insurance, and CO homeostasis. It also easily explains heterochiasmy just considering the different male and female SC lengths. This mechanism is expected to be conserved in other species.

## 1 Introduction

### 1.1 Crossing over interference: a short historical perspective

In sexually reproducing organisms, parental alleles are distributed in the offspring following the laws of segregation defined by Gregor Mendel more than 150 years ago (Mendel, 1866; Birchler, 2015). The chromosomal theory of heredity proposed by Thomas H. Morgan (1926) explains how new chromosomic (genomic) combinations are formed during the meiotic and fertilization processes. During meiosis, new chromosomes are formed using recombination between parental DNA molecules. The junctions between parental chromosomes named crossing-overs correspond to the cytologically observed chiasma defined by Frans A. Janssens (Janssens, 1909) (translated into English by Koszul and Zickler, 2012). Using *Drosophila*, Alfred Sturtevant genetically defined that one CO in a genetic interval reduces the probability of observing another CO in the adjacent interval (Sturtevant, 1913; Sturtevant, 1965), a phenomenon Hermann J. Muller coined *‘*CO interference’ in 1916 (Muller, 1916). John B.S. Haldane explained the genetic mechanism of interference by the statistical non-Poisson distribution of chiasmata observed in bivalents of various Angiosperms (Haldane, 1931). The correspondence between genetic crossing-overs (CO) and cytological chiasmata was elegantly demonstrated in maize (Creighton and McClintock, 1931; Coe and Kass, 2005). Various models of CO formation have been developed (reviewed in Haber, 2008; Gray and Cohen, 2016; Chuang and Smith, 2023), including various models trying to explain CO interference, ever since Haldane (Berchowitz and Copenhaver, 2010; Sun et al., 2017; Otto and Payseur, 2019; Saito and Colaiácovo, 2019; Chuang and Smith, 2023).

In this minireview, we will discuss what we have recently learned from plants regarding the integrated mechanisms regulating synapsis, CO interference, CO insurance, CO homeostasis, and heterochiasmy.

### 1.2 An integrated molecular model for CO formation in plants

**FIGURE 1:**
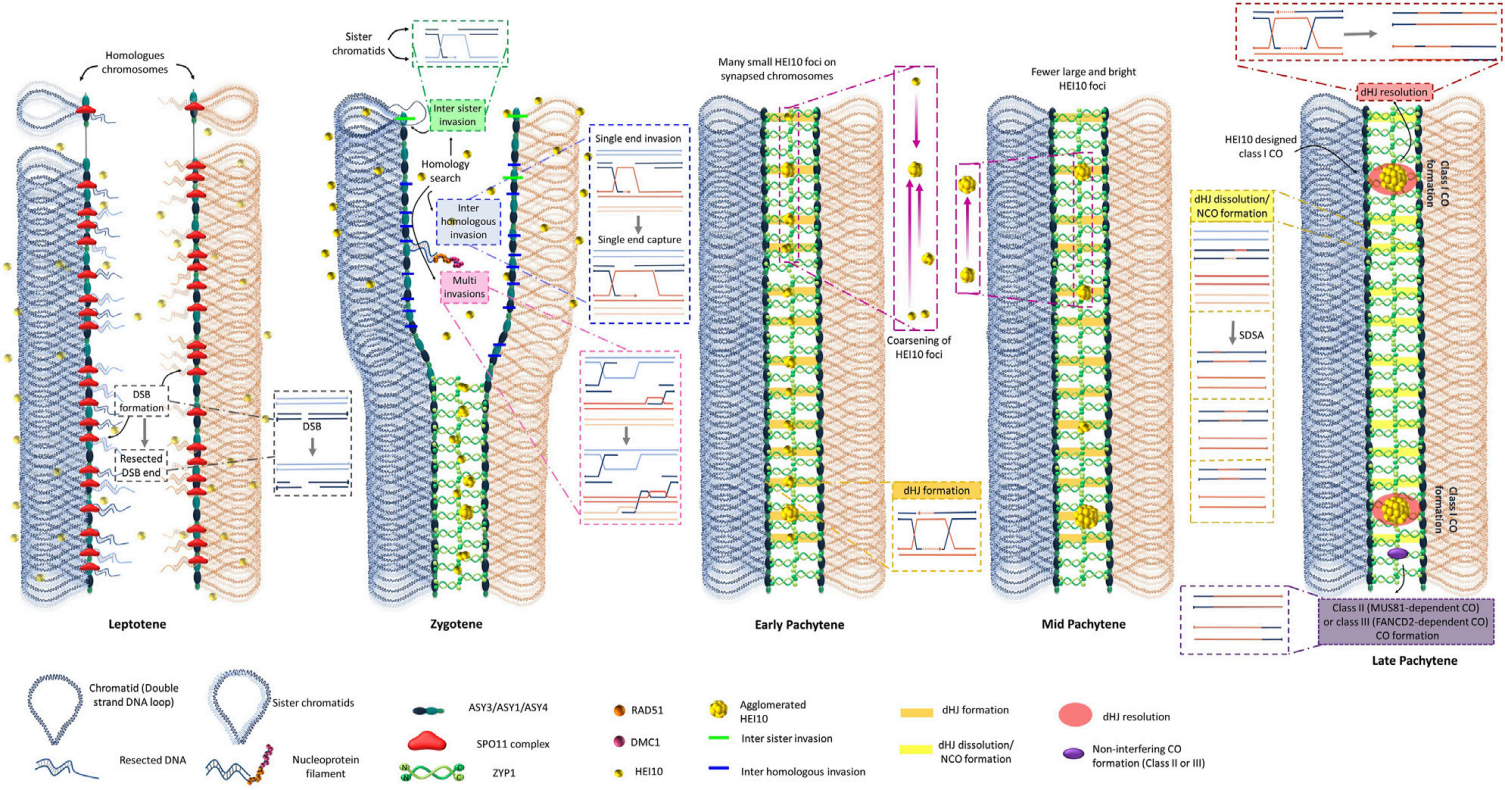
An integrated model explaining CO interference. Schematic representation of the formation of CO in just a pair of homologous chromosomes. To simplify, the global arrangement of the chromosome is depicted with two different colors for the homologs (red and blue) and two different tones for sister chromatids (light red and light blue). At this scale, each double-stranded DNA molecule is depicted as a simplifying line. The molecular steps necessary to understand the molecular mechanism of CO formation are depicted in magnified insets, where the 5′ ends of each DNA strand are represented by line with extremities with dots. **In leptotene,** the replicated chromosomes are represented with their important features for meiotic recombination. The axial elements (AE) including ASY1, ASY3, and ASY4 are assembled at the base of the loop of two sister chromatids attached by meiotic cohesins including REC8. Establishment of the proteinaceous axial element is essential to form the Synaptonemal Complex (SC). The formation of tens of DNA **double-strand breaks (DSBs)** for each of the only two homologous chromosomes pictured is processed by the evolutionarily conserved SPO11 complex. DSB ends are resected at either 5′ end to produce 3′ overhang, which are recognized by Rad51 and Dmc1 to generate inter-homologue strand-invasion. The 3′ end can invade either a homolog or a sister chromatid. In addition, multi-invasion of the 3′ tails on a homolog and/or sister chromatid is also possible to find an intact homologous template for HR repair. 3′ tail invasion in the homologous intact molecule generates a displacement loop (D-loop) through single end invasion (SEI). **In zygotene,** multiple SEI are used for homologous chromosome recognition/pairing. The transverse filaments ZYP1 of the synaptonemal complex (SC) start to polymerize at the center of the tripartite SC between two lateral elements. In **early pachytene**, the homologous chromosomes are fully synapsed from telomere to telomere. The SC allows the diffusion in only one dimension of the limiting CO factor HEI10 that can form coarsening recombination nodules explaining interference between CO class I (adapted from Morgan et al., 2021; Durand et al., 2022). In mid pachytene, various recombinant intermediates can be associated with HEI10 foci that progressively form larger agglomerated foci and eliminate the possibility of close CO design, except in the case of non-interfering COs that are not HEI10-dependant. **At the end of pachytene**, most of the chromosomal positions with SEI have not been transformed in CO but are resolved as NCO. At least one and generally few interferent (spaced out) COs are present on each bivalent, except where non-interfering (class II or class III) COs are formed.

The meiotic division leads to the reduction by half of the chromosome number in order to balance the fertilization process (Zickler and Kleckner, 2015). Meiosis is one of the most dynamic plant cellular processes (Ronceret and Pawlowski, 2010). It allows, after one step of replication, the equilibrated segregation of homologous chromosomes (meiosis I) followed by the segregation of sister chromatids (meiosis II) to produce four haploid spores (Mercier et al., 2015; Wang and Copenhaver, 2018; Gutierrez-Pinzón et al., 2021; Lloyd, 2023). During meiotic prophase I, homologous chromosomes undergo a genetically regulated process of recombination to create unique new chromosomes from two parental ones. The molecular models for meiotic CO formation of the last 40 years are based on the DNA Double-Strand Breaks (DSBs) Repair model of meiotic recombination proposed by Resnick, 1976 and refined by Szostak et al., 1983. This model explains how two DNA molecules can form a new recombined one using the error-prone repair mechanism of homologous recombination (HR). The basic mechanism of meiotic homologous recombination involves the formation of hundreds of programmed DSBs inflicted to the genome at the beginning of the leptotene stage (de Massy, 2013; Mercier et al., 2015; Yadav and Claeys Bouuaert, 2021). The number of DSBs varies from species to species: 150 to 250 in Arabidopsis (Vignard et al., 2007), 450 to 550 in maize (Pawlowski et al., 2003), and around 400 to 1,500 in hexaploid wheat (Benyahya et al., 2020). These various DSBs are formed in plants by the SPO11 complex containing SPO11-1 (Grelon et al., 2001; Shingu et al., 2012; Sprink and Hartung, 2014; Da Ines et al., 2020; Ku et al., 2020), SPO11-2 (Stacey et al., 2006; Benyahya et al., 2020; Fayos et al., 2020; Sprink and Hartung, 2021; Li et al., 2022; Hyde et al., 2023), MTOPVIB (Fu et al., 2016; Vrielynck et al., 2016; Xue et al., 2019; Jing et al., 2020; Steckenborn et al., 2023), PRD1 (de Muyt et al., 2007; Shi et al., 2021; Wang et al., 2022), PRD2 (de Muyt et al., 2009; Wang C. et al., 2023), PRD3 (de Muyt et al., 2009; Lambing et al., 2022; Wang et al., 2022), and DFO (Zhang et al., 2012). All these DSB factors form a complex where SPO11-1/2 (TOPVIA-like subunit) presents the catalytic transesterase (endonuclease) activity associated with MTOPVIB (TOPVIB-like subunit), underlining that the SPO11 complex conserve the structure of a DNA topoisomerase (Vrielynck et al., 2016; Vrielynck et al., 2021; Wang Y. et al., 2023). In this model, all original DSBs are not necessarily transformed into COs, but all COs are designed from a subset of DSBs (Figure 1). In plants, only a small subset of DSB sites is converted into COs (Mercier et al., 2015). Though in most species studied, the global genomic position of DSBs is randomly distributed along the length of chromosomes (He et al., 2017; Choi et al., 2018), it is known that at smaller scale, there are hotspots of DSBs in open chromatin regions near transcriptional start sites with low nucleosome density in Arabidopsis (He et al., 2017; Choi et al., 2018). It was recently shown that these DSB sites are also silenced to avoid recombination on transcribed genes (Wang et al., 2022). The number of DSBs can finally affect the genomic distribution of CO (Xue et al., 2018). The SPO11 complex is associated with axial elements (AE) of the synaptonemal complex (SC) including the HORMA domain ASY1/PAIR2 (Armstrong et al., 2002; Nonomura et al., 2006; Sanchez-Moran et al., 2007; Cuacos et al., 2021; Wang C. et al., 2023) as well as the ASY3/PAIR3/DSY2 (Wang et al., 2011; Ferdous et al., 2012; Lee et al., 2015) and ASY4 coiled-coil proteins (Chambon et al., 2018), supposably forming the basis of chromatin loops (Vrielynck et al., 2021; Lambing et al., 2022) (Figure 1 Leptotene stage).

The process of meiotic recombination is realized in a context of replicated chromosomes. Each DSB can therefore be repaired by homologous recombination using different undamaged homologous sequences as a repair template. The sites where the identical sister chromatid is used as the repair template form non-CO events (Figure 1). The sites where the DSB repair is processed using either one of the two polymorphic DNA sequences of the homologous chromosomes as the repair template can enter the pathway to form COs (Figure 1). During meiosis, a specific cohesin complex is installed between sister chromatids and contains REC8 instead of SCC1 (Chelysheva et al., 2005; Golubovskaya et al., 2006; Shao et al., 2011) as well as SMC1/3 (Lam et al., 2005; Bolaños-Villegas, 2021), SCC2 (Wang et al., 2020), SCC3 (Chelysheva et al., 2005), CTF7 (Bolaños-Villegas et al., 2013), and PDS5 (Pradillo et al., 2015). The putative helicase MCM8 (Crismani et al., 2013) and the cohesion-like SMC5/6 complex that favors sister chromatin HR repair in somatic cells are also tightly controlled during meiosis to allow the activity of interhomolog HR (Watanabe et al., 2009; Liu et al., 2014; Knoll et al., 2014; Chen et al., 2021; Zhu et al., 2021; Jiang et al., 2022). These DSBs are readily resected 5′ to 3′ by the exonuclease activity of MRE11 to form single-strand overhang 3′ extremities (Puizina et al., 2004; Ji et al., 2013) and allow the liberation of SPO11-bound oligonucleotides (Choi et al., 2018). MRE11 forms a complex with RAD50 (Bleuyard et al., 2004), NBS1 (Akutsu et al., 2007; Waterworth et al., 2007), and COM1 (Uanschou et al., 2007; Ji et al., 2013; Wang et al., 2018). These 3’ free single-strand DNA extremities are protected by various RPAs (Chang et al., 2009; Osman et al., 2009; Li et al., 2018; Aklilu et al., 2020), replaced by recA type recombinases RAD51 (Bleuyard et al., 2005; Da Ines et al., 2022; Li et al., 2022), RAD51C (Bleuyard et al., 2005; Jing et al., 2019), XRCC3 (Bleuyard and White, 2004; Zhang et al., 2015), and DMC1 (Couteau et al., 1999; Deng and Wang, 2007; Kurzbauer et al., 2012; Wang et al., 2016; Colas et al., 2019; Szurman-Zubrzycka et al., 2019). BRCA2 directly interacts with RAD51 and DMC1 and could facilitate their loading on the resected end of DSBs (Dray et al., 2006; Fu et al., 2020). These recombinases have the property to form nucleoprotein filaments that can bring together the single-strand DNA extremities with its specific homologous double-strand DNA molecules in a process known as single-end invasions (SEIs) (review in Emmenecker et al., 2022). Different recombinase subcomplexes exist and have differential functions between inter-sister SEI and interhomolog SEI (Kurzbauer et al., 2012; Pradillo et al., 2012; Su et al., 2017). The main biochemical difference between the Arabidopsis RAD51 and DMC1 recombinases tested *in vitro* is that RAD51 pairing activity seem not to be influenced by Ca^2+^ while DMC1 pairing activity is greatly enhanced in the presence of Ca^2+^ (Kobayashi et al., 2019). The most recently supported model proposes a symmetrical loading of RAD51 and DMC1 homotypic filaments on both DSB ends of a break (Da Ines et al., 2022). Interestingly, the role of the meiotic specific DMC1 seems to attenuate the strand exchange activity of the mitotic and meiotic RAD51 recombinase (Da Ines et al., 2022) (Figure 1 Zygotene stage). Interhomolog SEI is also promoted during meiosis by axial proteins such as ASY1 (Sanchez-Moran et al., 2007; De Muyt et al., 2009). Other recombinase accessory proteins modulate SEI (review in Emmenecker et al., 2022). For example, RAD54 assists the RAD51 interhomolog repair of meiotic DSB (Hernandez Sanchez-Rebato et al., 2021), while the meiotic specific HOP2 Vignard et al., 2007; Uanschou et al., 2013; Shi et al., 2019, Emmenecker et al., 2022) and MND1 (Kerzendorfer et al., 2006; Lu et al., 2020) form a complex that could assist DMC1 strand invasion (Kang et al., 2015). HOP2 could also prevent illegitimate recombination between non homologous chromosome regions (Farahani-Tafreshi et al., 2022) (Figure 1 Zygotene stages). In rice, HOP2 can directly interact with the ZEP1 SC central component (Shi et al., 2019).

Hundreds of DSB sites allow the formation of the corresponding hundreds of SEI sites along the zygotene homologous chromosomes (Figure 1 Zygotene stage). These multiple sites of SEI connections (known as joint molecules JMs), involving base pair DNA sequence homology recognition, are usually proposed to explain the mechanism used for the correct association (also known as pairing process) between the homologous chromosomes (Zickler and Kleckner, 1999; Pawlowski et al., 2003; Higgins et al., 2004). Most of these numerous SEIs will not form CO but are proposed to allow the accurate alignment between the right partners not only on a local scale but along the whole chromosome length. The excess of SEIs compared to the restricted final number of COs could therefore explain the accuracy of the pairing process between chromosome bivalents concomitant to synapsis (Gutierrez-Pinzón et al., 2021) (Figure 1 Zygotene stage). The recognized paired interaction zones are stabilized by the zipper-like proteinaceous structure known as the synaptonemal complex (SC). ZYP1 (ZEP1 in rice) is the central element forming a ladder between the two axial elements of the SC and connecting homologs from telomere to telomere (Higgins et al., 2005; Wang et al., 2010; Barakate et al., 2014; Capilla-Pérez et al., 2021; France et al., 2021) (Figure 1 Zygotene stage). The various SEI sites form regions of DNA triple helices known as Displacement-loops (D-loops). Most D-loops do not enter a CO pathway and several anti-recombinase pathways (described later) can dissociate the invading strand from the D-loop and re-anneal with the other DSB end, leading to small patches of hybrid DNA known as non-Crossover (NCO). The dissolved D-loops are proposed to be repaired via the Synthesis-Dependent Strand Annealing (SDSA) model during meiosis (Allers and Lichten, 2001; Puchta, 2005; Vu et al., 2017; Wang and Copenhaver, 2018) (Figure 1 Zygotene and pachytene stages).

For the few D-loops that enter a CO pathway, the invading strand is extended by DNA polymerases that realize a local DNA synthesis using the undamaged strand to copy it (Figure 1 Zygotene stage). The displaced strand of the undamaged DNA molecule hybridizes with the other 3’ end of the DSB in a process known as second end capture (Figure 1 Zygotene stage). These joint molecules can interchange part of their homologous strands extending the hybridization zone in a process known as branch migration that leads to the formation of double Holliday junctions (dHJs) (Figure 1 Pachytene stages). The asymmetric resolution of these dHJs can lead to the formation of COs that covalently link new portions of two homologous chromosomes (Szostak et al., 1983).

Two principal classes of CO pathways, one sensitive to interference (class I) and the other insensitive to interference (class II), have been defined in *S. cerevisiae*, animals, and plants (Mercier et al., 2005; Holloway et al., 2008; Gray and Cohen, 2016).

The major pathway (accounting for around 85% of COs in Arabidopsis) is called class I and is subject to CO interference. The formation of the class I CO involves members of the ZMM pathway composed of ZIP4 (Chelysheva et al., 2007; Shen et al., 2012), MER3 (Mercier et al., 2005; Wang et al., 2009), HEI10 (Chelysheva et al., 2012; Wang K. et al., 2012), PTD (Wijeratne et al., 2006; Ren et al., 2019), SHOC1/ZIP2 (Macaisne et al., 2008; Ren et al., 2019), mutS-like MSH4 (Higgins et al., 2004; Zhang et al., 2014b), mutS-like MSH5 (Higgins et al., 2008a; Luo et al., 2013), mutL-like MLH1 (Dion et al., 2007; Liu et al., 2022), and mutL-like MLH3 (Jackson et al., 2006; Colas et al., 2016; Mao et al., 2021). The mut-Lγ resolvase (formed by the MLH1-MLH3 heterodimer of the ZMM complex) can potentially stabilize D-loop structures and mature them as CO (review in Ziolkowski, 2023). This interfering pathway also requires the leading strand DNA polymerase epsilon (Ronceret et al., 2005; Huang et al., 2015; Wang et al., 2022a; Wang et al., 2022b) as well as the potential lagging strand DNA polymerase POLD1 (Wang et al., 2019) and RFC1 factor (Wang Y. et al., 2012) probably necessary for the synthesis steps of HR. This interfering pathway is limited by the HCR1 encoding a Protein Phosphatase X1 that can interact with HEI10, PTD, MSH5, and MLH1 (Nageswaran et al., 2021). In rice, a new plant specific ZMM member named HEIP1 interacting with HEI10, ZIP4, and MSH5 was also identified (Li et al., 2018). In tetraploid wheat, MSH4/MSH5 mutants also demonstrate an 85%–15% proportion of the two CO pathways in wheat species (Desjardins et al., 2020). In hexaploid wheat, the classical Ph1 locus controlling pairing and recombination between homeologous chromosomes, which is very important for breeding strategies, corresponds to a ZIP4 homolog (Rey et al., 2017).

The second minor non-interfering pathway (accounting for 15% of COs in Arabidopsis, around 10% of COs in rice) is called class II and involves the MUS81 protein (Berchowitz et al., 2007; Higgins et al., 2008b; Geuting et al., 2009). The MUS81 complex has a highly controlled endonuclease activity acting on selective DNA structures, such as D-loop and HJ, and can act on several replication and recombination intermediates during mitosis and meiosis depending on its interacting partners and phosphorylation status (Pfander and Matos, 2017). In rice, GEN1, the homolog of the Holliday junction resolvase is necessary for class II CO formation (Wang et al., 2017) while it is not in Arabidopsis (Bauknecht and Kobbe, 2014; Olivier et al., 2016), suggesting that the MUS81 pathways have diverged between dicots and monocots. Compared to Arabidopsis, the MUS81 pathway contributes even less to CO designation in rice (Wang et al., 2017; Mu et al., 2022), suggesting that the weight of the different pathways described in this review probably depend on each species.

In Arabidopsis, a second non-interfering pathway of CO, depending on FANCD2 and parallel to the MUS81 non-interfering CO pathway, contributes to the formation of some type II COs (Kurzbauer et al., 2018). The FANCD2 pathway, but not the MUS81 pathway, affects the distribution of class I CO (Li et al., 2021). In order to avoid confusion, this distinct FANCD2 pathway could be designed as the non-interfering class III CO pathway (Gutierrez-Pinzón et al., 2021).

Various meiotic interhomolog intermediate dissolution pathways (or anti-recombinase pathways) have been identified in Arabidopsis using meiotic mutant suppressor screens restoring fertility. Double mutant and cytological marker analysis have shown that they all reduce specifically the MUS81 dependent non-interfering class II CO pathway. These anti-recombinase pathways act using parallel mechanisms since mutants combining the different pathways lead to additive massive meiotic non-interfering CO formation (Crismani et al., 2012; Girard et al., 2015; Séguéla-Arnaud et al., 2015; Serra et al., 2018; Singh et al., 2023).

The first anti CO pathway requires the RECQ4A and RECQ4B helicase activity homolog to mammal BLM and yeast SGS1 (Hartung et al., 2007; Higgins et al., 2011; Séguéla-Arnaud et al., 2015; Serra et al., 2018). Combine with TOP3α (Hartung et al., 2008) and RMI/BLAP75 (Chelysheva et al., 2008), this complex is known as the RTR complex in plants and is similar to the ‘dissolvosome’ BTR complex in mammals and the yeast STR complex (Emmenecker et al., 2022). In Arabidopsis, recq4A mutant suppresses CO dependent on MUS81 activity (Séguéla-Arnaud et al., 2015). The RECQ4A protein is found on recombination intermediates of telomeres during meiosis and could particularly restrict CO class II on these chromosomal regions (Higgins et al., 2011). *Recq4* mutants increase by around three times the number of COs in rice, tomato, and pea (Mieulet et al., 2018). It seems that this RTR complex has functionally diverged between Arabidopsis and tomato (Whitbread et al., 2021).

The second anti CO class II pathway requires FIDGETIN-like AAA-ATPase (FIGL1) (Girard et al., 2015) associated with FLIP (Fernandes et al., 2018). This FIGL1 is an antagonist to the BRAC2 recombinase (Kumar et al., 2019). In rice, the FLIP-like MEICA protein can interact with the TOP3α and MSH7 (Hu et al., 2017), suggesting coordination between the first anti CO pathway at least in rice. MEICA and FIGNL1 both have an anti CO activity affecting class II CO (Zhang et al., 2017; Yang C. et al., 2022). These pathways could also have different strengths in male *versus* female meiosis as observed by the male specific effect of the FIGL mutation in rice (Zhang et al., 2017).

The third anti CO pathway requires the Fanconi Anaemia (FA) pathway comprising the FANCM helicase (Crismani et al., 2012); its cofactors MHF1 and MHF2 (Girard et al., 2015); and the FANCC, FANCE, and FANCF subcomplex (Singh et al., 2023).

It is important to clarify that any of these three parallel anti-recombinase pathways, which substantially increase the number of class II CO, have been shown to abolish CO interference measured genetically. The interpretation of these confounding results is that the large number of class II COs, by decreasing the space between COs, mask the class I spacing mechanism expected to remain unaffected (Crismani et al., 2012; Girard et al., 2015; Séguéla-Arnaud et al., 2017; Fernandes et al., 2018; Li et al., 2021).

Interestingly, in Arabidopsis, FANCD2, FANCM, FIGL1, and RMI1 not only suppress non-interfering CO but also have a role in regulating the distribution of class I CO among chromosomes to insure at least one CO per bivalent (Li et al., 2021). In wheat, it was shown that FANCM not only suppresses class II non-interfering COs but also promotes class I interfering COs and insures at least one CO by bivalent (Desjardins et al., 2022).

It has been proposed that these dissolution pathways leading to NCO could be especially important for multi-invasion complexes formed between more than two DNA molecules that would otherwise lead to aberrant recombination intermediates (Emmenecker et al., 2022; Mu et al., 2022). Whether or not these pathways could be involved in a putative chromatid interference phenomenon (Zhao et al., 1995; Sarens et al., 2021) is still unknown.

In general, the combination of these different pro- and anti- CO pathways leads to the formation of only one or two COs per chromosome arm in plants, fungi, and animals (Otto and Payseur, 2019; Saito and Colaiácovo, 2019; Pazhayam et al., 2021; Tock et al., 2021; Lloyd, 2023).

However, despite the understanding of the molecular factors involved in CO formation in various model species, the mechanism by which COs tend to space out from each other has been debated for more than a century. The other mechanisms of the obligatory CO, CO homeostasis (where the variation of the initial number of recombination intermediate does not affect the final number of COs), and heterochiasmy (where CO rate is different between male and female meiosis) have also gained various insights from recent analysis in plant meiotic models.

### 1.3 A model of CO interference based on HEI10 coarsening along the SC compartment

**FIGURE 2:**
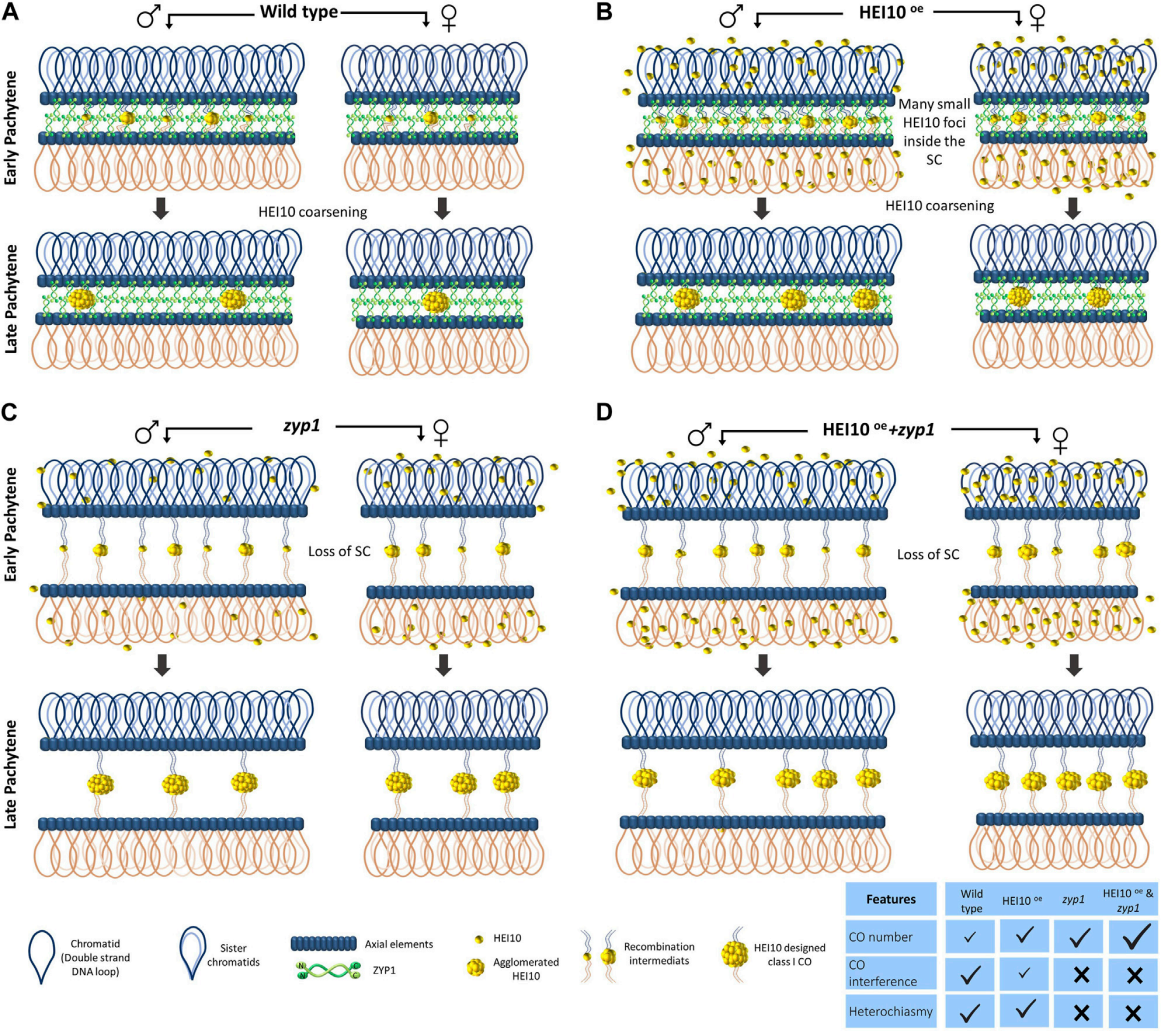
Many mechanisms of crossover interference can be explained by the HEI10 coarsening model along the ZYP1 synaptonemal complex structure. Schematic representation of the HEI10 localization at pachytene in *Arabidopsis thaliana* wild type **(A)**, *zyp1* mutant **(B)**, HEI10^oe^ overexpressed line **(C)**, and *zyp1*+ HEI10^oe^ **(D)** adapted from Durand et al., 2022. In wild type, HEI10 proteins are localized into foci. Many small HEI10 foci on synapsed chromosomes appear along the SC. With the progression of the pachytene stages, the number of HEI10 foci gradually decreases and few of them coarsen by agglomerating the smaller foci into the larger ones. Fewer foci are generated in females due to the shorter SC and leads to heterochiasmy **(A)**. In HEI10 OE, the coarsening process occurs as well as wild type and by keeping the heterochiasmy state, CO numbers in both sexes increase while CO interference decreases **(B)**. In the absence of SC, *zyp1*, unlike the presence of SC ones, free diffusion of HEI10 occurs in the nucleoplasm and abolish the competition among foci to absorb the HEI10 and all initial foci grow continuously by capturing the HEI10. Hence, this results in a loss of crossover interference and heterochiasmy but an increase in the CO number **(C)**. As it is expected, the combination HEI10 (OE) and *zyp1* provides larger increases in CO number than *zyp1* and HEI10 OE alone, and CO interference and heterochiasmy are abolished **(D)**.

Recently, several lines of evidence converged to pinpoint the fundamental role of the localization dynamics of the specific pro-crossover ZMM HEI10 factor on meiotic chromosomes to explain the CO interference phenomenon. HEI10 is a member of a family of RING-finger domain E3 ubiquitin ligase (De Muyt et al., 2014) including Zip3 in *S. cerevisiae* (Agarwal and Roeder, 2000), HEI10 and RNF212 in mammals (Ward et al., 2007; Kong et al., 2008), Vilya in *Drosophila* (Lake et al., 2015) and ZHP-3 in *C. elegans* (Bhalla et al., 2008; Zhang et al., 2018). In Arabidopsis, the expression of HEI10 responds similarly to its overlapping adjacent gene MRD1 to pathogen inoculations via the OZF1 transcription factor (Singh et al., 2022). AtHEI10 is also transcriptionally repressed by a Heat Shock Binding Protein (HSBP) that contributes to the adaptative CO formation in response to change in temperature (Kim et al., 2022) suggesting that HEI10 expression is responding to various environmental factors. In immunolocalization experiments, HEI10 foci show a peculiar pattern with a foci number progressively decreasing from late zygotene to diakinesis with late signal associated with other members of the class I CO such as MLH1 and MLH3 in Arabidopsis and rice (Chelysheva et al., 2012; Wang K. et al., 2012). It was noticed that HEI10 can show either small and faint numerous foci at the beginning of the zygotene stage or large and bright signals at the end of the pachytene stage in rice, Arabidopsis, and wheat meiocytes (Wang Y. et al., 2012; Duroc et al., 2014; Desjardins et al., 2020; Osman et al., 2021). Natural variation in this gene is associated with variation in meiotic recombination rate observed between Arabidopsis ecotypes (Ziolkowski et al., 2017). The dosage of HEI10, reduced in *hei10* heterozygotes or increased in HEI10 overexpression line is a determinant for CO number and the strength of CO interference (Ziolkowski et al., 2017; Serra et al., 2018; Morgan et al., 2021). Using super-resolution SIM track of the HEI10 immunolocalization signal on fully synapsed pachytene bivalents, it was observed that the intensity of HEI10 signal per foci depends on the total number of observed foci per bivalent, with brighter signal when unique foci are present on bivalents (Morgan et al., 2021). During early zygotene, faint HEI10 signals are uniformly distributed along the bivalents (Figure 1. Zygotene stage), the number of HEI10 foci progressively decreases during zygotene and pachytene stages with an aggregation of brighter and larger HEI10 foci at the expense of the fainter and smaller adjacent foci (Figure 1 Zygotene and Pachytene stages). Ultimately by late pachytene, few large bright foci have been spaced out by the mechanism of HEI10 coarsening that has depleted the surrounding HEI10 sites on bivalents (Figure Fig1. Late Pachytene stage). However, by which mechanism HEI10 can coarsen and how it is related to other ubiquitination processes during meiosis require further investigations (Orr et al., 2021; Ren et al., 2021). It is worth underlining that the analysis of pachytene bivalents using electron microscopy had already noticed different sizes and shapes of early (poorly interfering) *versus* late interfering **‘recombination nodules’ (RN)** localize along the synaptonemal complex (Anderson and Stack, 2005)*.* Morgan and colleagues proposed a mathematical ‘diffusion mediated HEI10 coarsening model’ where the SC is seen as a linear structure allowing the diffusion of the HEI10 signal in only one dimension (Morgan et al., 2021). This model convincingly predicts differently tested situations in WT and *hei10* mutant or HEI10^oe^ overexpression line (Morgan et al., 2021) (Figure 2). The model explains very well how the HEI10 recombination nodules become progressively distant from each other. This model also implicitly explains how the mechanism of interference is not affected by the formation of either class II MUS81 dependent CO, nor class III FANCD2 dependent CO, though either of the three pathways can use the same SEI initial recombination intermediates. It also suggests that the remaining SEI sites without HEI10 bright foci are transformed into NCO or non-interfering COs (Figure 1 Pachytene stages).

The coarsening model was initially proposed in *C. elegans*, whose HEI10 homolog ZHP-3 has a similar behavior (Bhalla et al., 2008; Zhang et al., 2018; Zhang et al., 2021). The SC also seems to have peculiar liquid-crystal properties that can facilitate long range signal transduction (Rog et al., 2017). Whether or not similar properties of the plant SC could help the progressive coarsening of HEI10 remains to be determined in plants.

The situation in mammals could be similar since the HEI10 accumulates at designed CO sites (Qiao et al., 2014). However, in mammals, the interference mechanism might not involve the HEI10 protein itself but rather its antagonist RNF212 SUMO ligase (Qiao et al., 2014; Rao et al., 2017). Indeed, in mammals, RNF212 seems to be the limiting factor for CO class I design since it has sequence variants explaining the recombination rate in human populations (Kong et al., 2008) and shows a sensitive dosage role during mouse meiotic recombination (Reynolds et al., 2013).

### 1.4 The SC/HEI10 coarsening could also explain the mechanisms of obligatory CO and CO homeostasis

The mechanism by which at least one CO is formed on each bivalent was recognized as essential to segregate the full set of homologous chromosomes during the meiotic reductional division (Darlington and Dark, 1932). This obligate CO was defined by Owen in 1949 (Owen, 1949). This ability to ensure CO (also known as **CO insurance**) on each bivalent can easily be integrated into the HEI10 coarsening model along the SC. The HEI10 coarsening model assumes that if enough HEI10 is deposited on a bivalent, it will produce at least one CO per bivalent explaining CO insurance.

The mechanism of CO homeostasis was defined by Martini et al., 2006 to explain a situation observed in budding yeast where the variation of the number of DSB (i.e., 80% reduction) does not significantly affect the final number of COs. In Arabidopsis, a 30%–40% reduction in DSB number leads to a proportionate though smaller reduction of CO number, affecting their distribution and redirecting them toward the telomeres (Xue et al., 2018). In maize though, it appears that CO homeostasis is somehow limited (Sidhu et al., 2015). Anyway, the coarsening HEI10 model could also account for CO homeostasis if we imagine that a DSB-independent limiting amount of expressed HEI10 per meiocyte limit the final number of the major class I CO. The large excess of DSB and SEI intermediates compared to the final CO number could explain how the bright and large HEI foci designing class I COs might not be affected by a change in the number of DSB and earlier SEI intermediates, as long as the number of these intermediates is at least the same as or exceeds the number of final COs.

### 1.5 Role of synapsis in interference, obligate CO and heterochiasmy

In species with different CO rates between male and female meiosis (heterochiasmy), the length of SC is particularly different as well. SC length also depends on chromosome size and the SC length is proportional to CO rate (Zickler and Kleckner, 2015).

As already mentioned, the ZYP1 is the central element of the SC. In contrast to the *S cerevisiae*, Zip1 homolog is considered a member of the ZMM pathway that is indispensable for synapsis and CO formation (Sym et al., 1993), the mutation of *zyp1* in Arabidopsis and rice disprupts synapsis but allows the formation of a higher number of COs per meiosis (Wang et al., 2010; Capilla-Pérez et al., 2021; France et al., 2021). It appears that this uncoupling of SC and CO class I formation in plants fortunately allowed the role of the SC in the interference mechanism to be refined. In addition, plants do not have a bone fine pachytene checkpoint leading to apoptosis when bivalents cannot form (De Jaeger-Braet et al., 2022), allowing easier observations of the consequences of the *zyp1* mutation than in animals or budding yeast. In the absence of *zyp1*, the number of COs increases, but interference, obligatory CO, and heterochiasmy are lost (Capilla-Pérez et al., 2021; France et al., 2021). In *zyp1* mutants, the HEI10 foci are still formed despite the loss of the SC and the formation of bright HEI10 signals (Capilla-Pérez et al., 2021), suggesting that the HEI10 diffusion still occurs but in three dimensions in the nucleoplasm, instead of being canalyzed by the SC on each bivalent (Figure 2C). So when synapsis is lost, heterochiasmy is also lost. This data is interpreted as the fact that heterochiasmy does not rely on peculiar regulation of the RH machinery itself except the one imposed by the SC length. It is predicted that all the factors affecting longer SC length will increase CO rate; the one decreasing SC length will decrease CO rate.

The HEI10 coarsening model proposed by Morgan et al., 2021 also reliably accounts for these observed facts in male and female meiosis of Arabidopsis (Durand et al., 2022). The longer male SC can integrate more HEI10 and have a longer distance to space them out, leading to both a higher recombination rate and higher interference (Figure 2B). Finally the HEI10 coarsening model also predicted as well the combination of *zyp1* with HEI10^oe^ situations (Durand et al., 2022; Fozard et al., 2023) (Figure 2D). The situation is also observed in a new separation of function *zyp1* allele (Yang S. et al., 2022). ASY1 also mediates CO insurance and interference (Lambing et al., 2020; Pochon et al., 2022). The effect of ASY1, one of the axial elements of the SC, on CO and interference might also be partly due to the absence of ZYP1 installation in this mutant.

A previous model known as the beam-film model has been extensively proposed to explain the interference mechanism by a redistribution of the mechanical stress that dissipates at the site of CO designation, impeding the formation of another CO nearby (Kleckner et al., 2004; Zhang et al., 2014a; Otto and Payseur, 2019). The beam-film model and the HEI coarsening model are possibly not exclusive and the mechanisms of HEI10 diffusion or coarsening could be influenced by a mechanical stress potentially perceived through SC remodeling (Lambing et al., 2020; Yang C. et al., 2022).

Most of the meiotic studies are still made in male meiocytes but immunolocalization techniques used to analyze female meiocytes have now been developed (Escobar-Guzmán et al., 2015; Gordillo et al., 2020) in order to better understand heterochiasmy and identify the basis of the regulation of the male and female SC length difference.

## 2 Conclusion

Forty years after the DSB repair model (Szostak et al., 1983), the molecular pathways and factors leading to the formation of CO have been described in various fungi, animal, and plant models. An integrated model for understanding the CO patterning at the whole genome level remained more difficult to establish. The model reviewed here requires the observed excess of DBS sites for correct pairing between homologs and synapsis. The excess SEI sites are also required for interference and the progressive design of COs along bivalents that lead to the restricted number of final COs. The SEI sites unused to form CO are still repaired and explain the presence of NCO.

In the model reviewed here, which was initially proposed in *C. elegans* by Zhang et al., 2021 as well as in Arabidopsis by Morgan et al., 2021 and refined by Durand et al., 2022 and Fozard et al., 2023, the role of the synaptonemal complex SC, an evolutionary well conserved structure only present during meiosis, is clearly assigned: it allows the HR machinery to work on the bivalent as a unit separated from the rest of the nucleoplasm. This explains how SC is tightly interconnected with the homologous recombination process during meiosis. The SC can therefore restrict the diffusion of important recombination regulators such as HEI10 on a linear basis and explain the observed coordination of mechanisms controlling CO insurance, homeostasis, and interference. In this model, heterochiasmy relies on different SC lengths, as observed cytologically in various species (Zickler and Kleckner, 1999).
